# Geospatial disparities in adolescent mental health resources: school- and community-level evidence from Travis County, Texas

**DOI:** 10.3389/fpubh.2026.1845379

**Published:** 2026-07-06

**Authors:** Sharon V. Munroe, Jingjing Gao, Gayla Ferguson, Muinat Abolore Idris, Eric C. Jones

**Affiliations:** 1 Department of Health Promotion and Behavioral Sciences, School of Public Health, The University of Texas Health Science Center at Houston, Houston, TX, United States; 2 Department of Management, Policy and Community Health, University of Texas Health Science Center at Houston School of Public Health, Houston, TX, United States; 3 Department of Environmental and Occupational Health Sciences, University of Texas Health Science Center at Houston School of Public Health, Houston, TX, United States; 4 Department of Epidemiology, The University of Texas Health Science Center at Houston, School of Public Health, Houston, TX, United States

**Keywords:** adolescent mental health, geospatial analysis, health equity, school social work, school-based mental health services, social determinants of health

## Abstract

**Background:**

Adolescents in the United States face growing mental health challenges, with nearly one in three high school students reporting poor mental health in the past 30 days. Schools increasingly serve as key access points for mental health care. However, geographic disparities in access to school-based mental health services remain underexplored.

**Purpose:**

This study examines the spatial distribution of adolescent mental health resources in Travis County, Texas, and evaluates how school- and community-level factors are associated with the presence of on-site school social workers.

**Methods:**

School locations, enrollment data, and the presence of on-site social workers were mapped and analyzed alongside ZIP Code Tabulation Area-level indicators of community vulnerability using ArcGIS Pro. Additionally, data from public high school districts, community mental health clinics, and social determinants of health indicators were incorporated for geospatial analysis through spatial regression modeling in R.

**Results:**

Many high schools were located more than two miles from a free or low-cost mental health clinic, especially in the eastern regions of the county. Among 24 Travis County high schools, only 29% had on-site social workers. Spatial regression results showed that schools located in communities with higher poverty, unemployment, and limited English proficiency were significantly less likely to have an on-site social worker (*p* < 0.05). In contrast, schools in areas with lower education levels, more residents with disabilities, and high housing cost burdens were more likely to have on-site social workers (*p* < 0.05).

**Conclusion:**

Geospatial disparities in mental health service access represent an important challenge for high school students in Travis County. Public resources, primarily from local stakeholders, should be directed toward high school students in these communities. School-based social workers and telehealth partnerships help address some of these gaps. The findings emphasize the importance of equity-focused planning to provide accessible, school-centered mental health services.

## Introduction

Poor mental health among high school students is linked to serious challenges and risks, including depression, suicidality, academic and social challenges, gun violence, and long-term health issues (1–6). In 2022–2023, approximately 19.2% of United States (U.S.) adolescents and 17.3% of Texas adolescents aged 12–17 had a major depressive episode in the past year, according to the 2023 National Survey of Drug Use and Health (38) estimates. Capturing the first signs of poor mental health or symptoms among adolescents is critical, as an estimated 50% of all lifetime mental illnesses begin by age 14, and 75% begin by age 24 (7, 8). Also, adolescents showing signs of poor mental health but without a diagnosis of a mental disorder may face a higher risk of poor mental health and reduced quality of life as they transition to adulthood. Since 2020, the Texas Education Agency (TEA) has been working to support Texas schools in improving access to student mental and behavioral health as part of TEA’s Statewide Plan for Student Mental Health (41).

Social workers may serve as vital support resources for high school students dealing with common mental health challenges, such as depression and anxiety, based on experiencing elevated levels of stress or trauma (9–14). Receiving mental health services on campus is particularly crucial during major adolescent life changes or recent trauma. While social workers alone do not diminish stigma, their programming and efforts can boost the school’s ability to address students’ behavioral health more proactively and help reduce the stigma associated with discussing mental health challenges among students and staff. Additionally, school social workers often lead or co-facilitate prevention programs and school-wide interventions that teach coping skills, bolster family support systems, and increase staff awareness of adolescent mental health. By establishing collaborative networks, social workers can help remove barriers to accessing mental healthcare. Despite these benefits, their availability varies widely across school districts, often reflecting broader structural inequalities in funding and resource allocation (2, 9, 10, 15). While other school-based professionals, such as counselors and psychologists, also contribute to student mental health support, social workers are uniquely positioned to address both individual and community-level factors of health through case management and systems-level interventions.

Regarding Texas, the provision of adolescent mental health services in schools has expanded in recent years. Since 2020, the Texas Education Agency has supported schools in improving student mental and behavioral health access through its Statewide Plan for Student Mental Health (2024). Travis County, Texas, which includes Austin and nearby cities such as Del Valle, Pflugerville, Manor, Lakeway, and Westlake, has approximately 34,161 students in grades 9–12 enrolled across 34 public and private high schools (16). The county faces a shortage of mental health providers, with one licensed professional counselor per 674.6 people and one psychiatrist per 4,660, contributing to service “deserts” where psychologists or therapists are not readily available through nearby free or low-cost clinics (17, 18). Additionally, although some neighborhoods and schools in Travis County are served by Capital Metro bus routes, many require travel distances exceeding two miles, posing potential transportation barriers.

School-based mental health resource allocation is shaped not only by community need but also by policy and funding structures at the local and state levels (19, 20). In Texas, initiatives led by the Texas Education Agency aim to expand access to student mental health services; however, implementation varies across districts due to differences in funding capacity, workforce availability, and competing institutional priorities. These structural factors may contribute to the uneven distribution of school-based mental health personnel, even among communities with similar levels of need.

Additional local indicators suggest substantial unmet mental health needs among adolescents in Travis County (21). Data from Central Texas and systems serving the region indicate increasing demand for youth mental health services, including rising reports of anxiety, depression, and school-related behavioral health concerns. For example, high proportions of students eligible for free or reduced-price lunch and elevated absenteeism rates in certain districts further suggest concentrated areas of need (22, 23). At the same time, limited capacity within publicly funded programs and workforce shortages contribute to gaps between the need for services and their availability. These patterns provide important context for assessing whether the geographic distribution of school-based and community mental health resources aligns with underlying demand among adolescents in Travis County.

Although prior reports and descriptive mapping efforts have documented disparities in mental health resources, few studies have applied spatial analytic methods to examine how school-level and community-level factors jointly shape access to services. Descriptive approaches can show where services are located, but do not assess whether they are equitably distributed relative to need or how structural conditions influence their placement. Spatial methods allow for the identification of geographic clustering, spatial dependence, and mismatches between service availability and population vulnerability, providing a more rigorous understanding of inequities beyond simple visualization. This study focuses on Travis County, Texas, a rapidly growing and demographically diverse region with documented shortages of mental health providers, making it a relevant setting to examine disparities in adolescent mental health infrastructure. We use spatial analysis to both visually map the distribution of high schools, social workers, and community clinics and to quantitatively evaluate how neighborhood characteristics and school factors are associated with the presence of on-site social workers. By integrating mapping with spatial regression, this study provides a more comprehensive assessment of how place-based and structural factors shape access to school-based mental health support. This study is guided by a Social Determinants of Health perspective, which posits that structural and community-level conditions, such as socioeconomic disadvantage, demographic composition, and resource constraints, influence the distribution of institutional resources and access to services (24–26). Within this framework, neighborhood disadvantage may shape school funding environments, policy priorities, and staffing decisions, thereby affecting the availability of school-based mental health personnel and proximity to community-based care. By explicitly linking community context to resource allocation and spatial access, this approach provides a theoretical basis for understanding how geographic and structural inequities in adolescent mental health services emerge. This study addresses the following research questions:

(1) What is the geographic distribution of school-based and community-based mental health resources available to high school students in Travis County?
(2) How are school-level and community-level characteristics associated with the presence of on-site school social workers?

## Methods

### Study area

In the past two decades, the Austin Metropolitan Area, including Travis County, has been one of the fastest-growing areas within Texas and the United States (39). It is home to the state capital, the University of Texas at Austin, large employers, several school districts, and major hospitals and health clinics. It has an area of 994.05 square miles, and as of July 2024, the population was estimated to be 1,363,767 with 561,491 households (39).

### Data sources and measures

#### School-level data

ArcGIS Online was the source of several base maps and shapefiles for state, county, population, and highway data. Data on school locations, enrollment, and school characteristics were obtained from publicly available administrative datasets, including Austin Texas Data, 2024, and the U.S. Census Bureau, 2024. To determine whether high schools have social workers on-site in high schools required web searching and a consultation with the Del Valle Independent School District director of counseling (phone conversation with Victoria Esparza-Gregory on March 14, 2025). Several Travis County school districts have funding and community partnerships that provide high schools with on-site social workers as staff, including Del Valle, Manor, and Pflugerville school districts (phone conversation with Victoria Esparza-Gregory on March 14, 2025).

Addresses for free or low-cost mental healthcare clinics found via web searching were added to another computer spreadsheet and geocoded by address. Completed Memorandum of understanding between school districts with Texas Child Health Access Through Telemedicine for telehealth services in schools came from the program website (42) and included participating Travis County high schools; Texas Child Health Access Through Telemedicine participant schools’ addresses were compiled in an Excel table and geocoded.

#### Community-level data

Sociodemographic characteristics at the ZIP Code Tabulation Area level were obtained from the American Community Survey 5-Year Estimates (2018–2022), accessed via the United States Census Bureau. Variables were extracted using the Centers for Disease Control and Prevention Social Vulnerability Index framework, which includes indicators such as poverty, unemployment, disability, education level, household composition, minority status, language proficiency, health insurance coverage, and housing burden. ZIP code-level data were used to match schools to the demographic context of the surrounding community.

### Spatial analysis

All spatial statistical analyses were conducted in R (version 4.2.3) (27, 28) using the spdep and spatialreg packages for spatial lag and error models. Data management and preprocessing were performed in Python (version 3.10) (29), including integration of school and sociodemographic datasets. ArcGIS Pro (version 3.2, Esri) was used for geographic visualization and spatial data processing, including geocoding, ZIP code boundary overlays, and thematic mapping (30). Spatial lag and spatial error models were adopted to evaluate the association relationships between school- and community-level characteristics and the presence of on-site social workers on campus, especially when the dependent variable in this study is binary “Yes” or “No” to the question of whether the campus has on-site social workers (31–33).

## Results

### Spatial visualization of mental health resources

**Figure 1:**
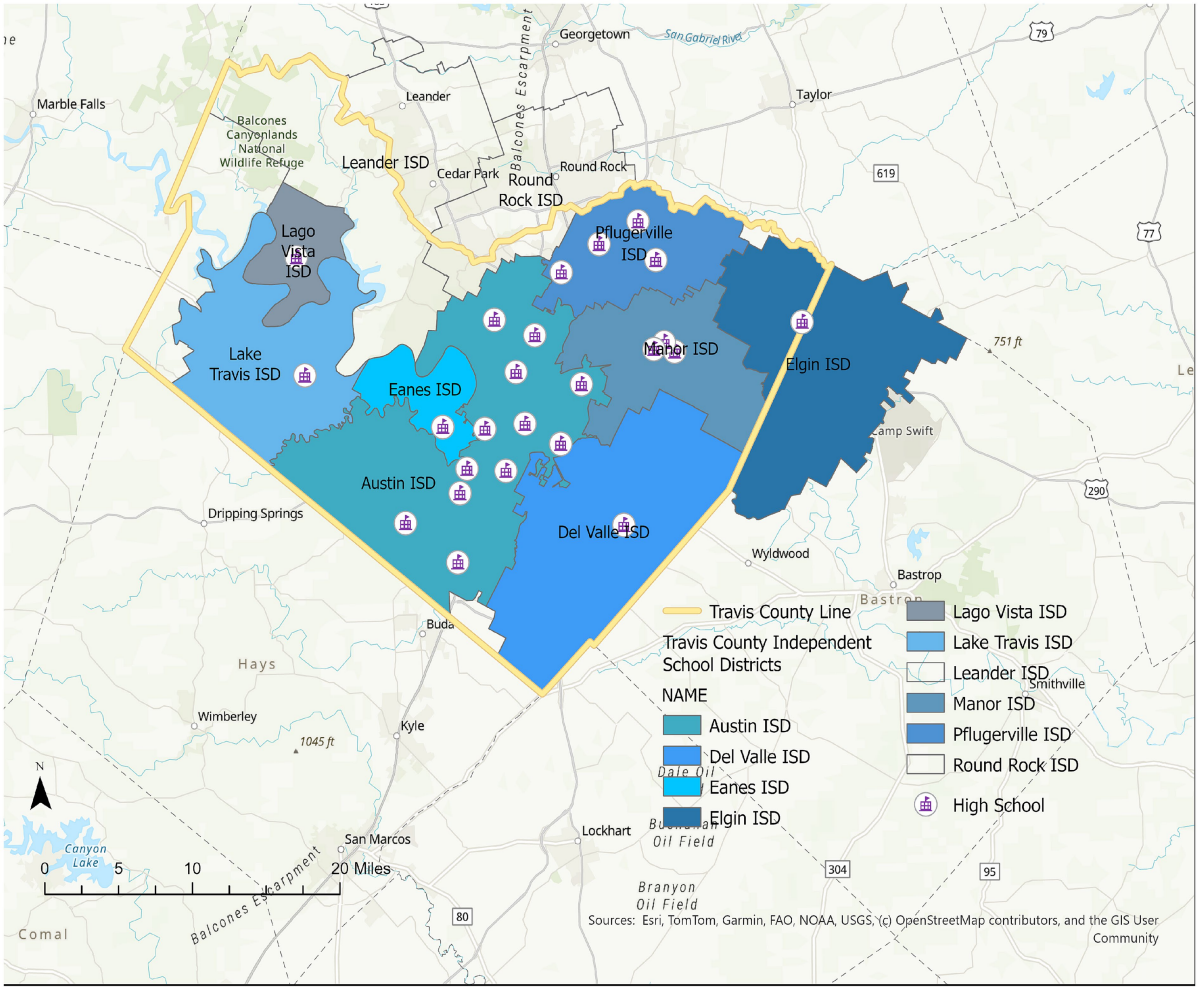
The Travis County line and its independent school districts (ISD). Those ISDs located at the county’s borders (e.g., Round Rock, Leander), some of which have Austin mailing addresses.

The study area of Travis County, one of the 254 counties in Texas, is highlighted in gold in Figure 1 (30). Travis County encompasses multiple school districts, and the city limits of municipalities such as Austin and Elgin do not entirely correspond with the county’s borders. Notably, public school districts in proximity to Travis County that share Austin mailing addresses include the Round Rock Independent School District and the Leander Independent School District, both located in Williamson County. Elgin High School, which serves the entire Elgin Independent School District, is situated within Travis County, near the boundary with Bastrop County. It is important to note that Elgin Independent School District’s elementary and middle schools are located in both Travis and Bastrop Counties (40).

**Figure 2:**
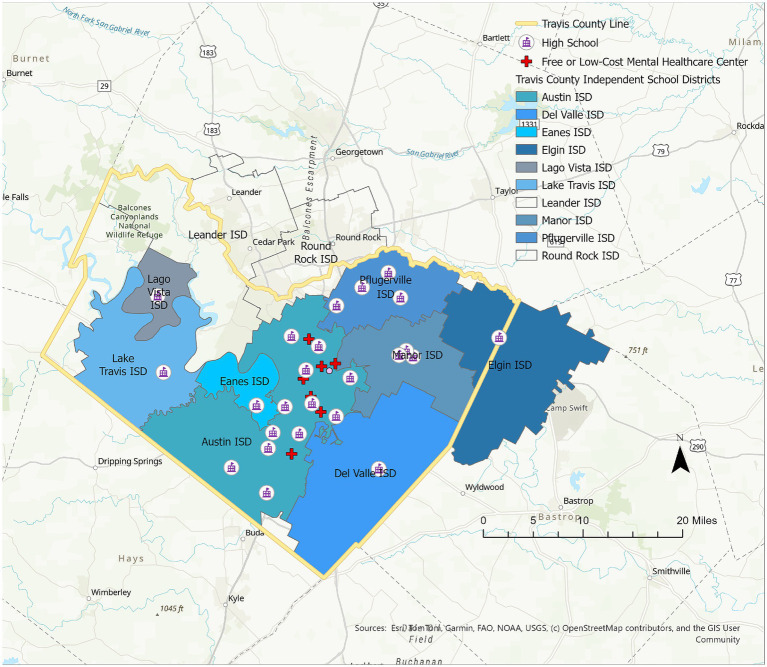
Geographic Distribution of Free or Low-Cost Community Mental Health Clinics and Their Proximity to High Schools in Travis County, Texas. Free or low-cost mental health clinics operated by Integral Care and other providers are mapped alongside the locations of public high schools in Travis County. The spatial distribution highlights that many high schools are situated more than two miles from the nearest clinic, with several located over five miles away, indicating potential gaps in accessible adolescent mental health services.

As shown in Figure 2, the locations of free or low-cost mental health clinics-including those managed by Integral Care-were geocoded and overlaid with high school locations in Travis County. The visual analysis reveals substantial geographic disparities in access: many high schools are located more than two miles from the nearest mental health facility, and in several instances, the distance exceeds five miles. These spatial patterns suggest limited accessibility of community-based mental health services for adolescents, particularly in districts such as Elgin, Manor, and Del Valle school districts, where on-campus support may be insufficient or unavailable.

**Figure 3:**
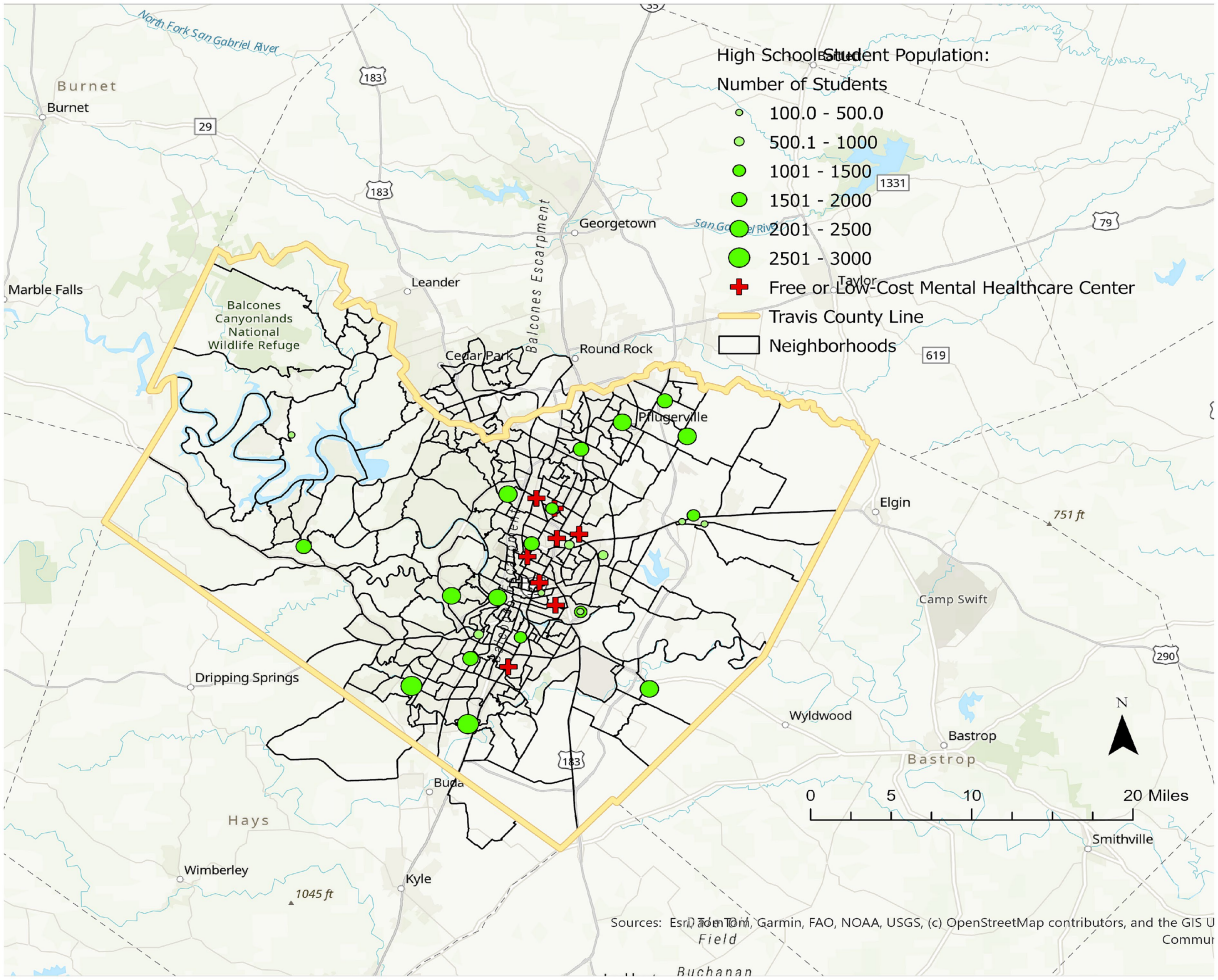
High School Student Enrollment and Proximity to Free or Low-Cost Community Mental Healthcare Clinics in Travis County, Texas. High schools are represented using graduated symbols corresponding to student enrollment size, while red crosses indicate the locations of free or low-cost mental health clinics. The map illustrates disparities in access, as several high schools with enrollment exceeding 2,000 students are located in neighborhoods with limited or no nearby low-cost mental health services.

Figure 3 presents the spatial relationship between high school student enrollment and the availability of free or low-cost community mental healthcare clinics in Travis County. Student population data were mapped using graduated symbols to represent school size, overlaid with neighborhood boundaries and clinic locations. The figure reveals that while some densely populated neighborhoods and large high schools-enrolling over 2,000 students-are located near clinics, many others are not. Several high-enrollment schools are situated in areas with limited or no proximal access to low-cost mental health services. These geographic gaps may present structural barriers to mental healthcare access for adolescents, particularly in underserved or rapidly growing neighborhoods. Additionally, demographic shifts in Travis County-including negative net migration reported in 2022-2023-may further complicate resource allocation and service delivery (U.S. Census, 2024a). Out-migration can reduce the tax base and workforce available to support local programs, while simultaneously increasing turnover in school enrollment and community networks. These dynamics may strain continuity of care, limit the stability of funding streams for mental health initiatives, and create challenges in planning service capacity across both schools and community clinics.

**Figure 4:**
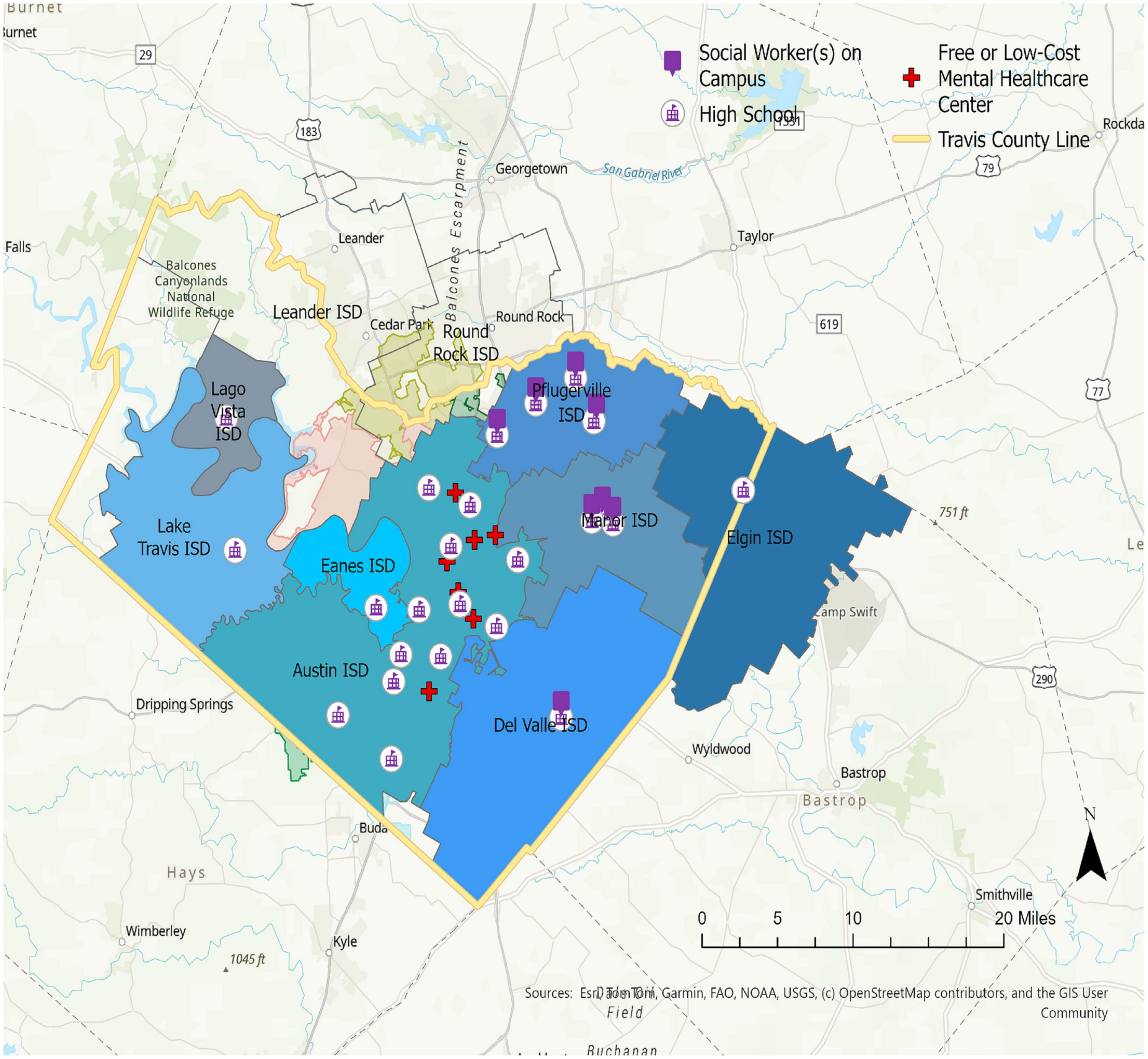
High Schools with On-Campus Social Workers and Proximity to Free or Low-Cost Mental Health Clinics in Travis County, Texas. This map displays high schools in Travis County that have on-campus social workers (purple squares), overlaid with high school locations (purple circles) and free or low-cost community mental health clinics (red crosses). Several schools with on-campus support are located in areas distant from mental health clinics, suggesting these staff fill essential service gaps, particularly in underserved or geographically isolated regions.

Figure 4 presents the distribution of high schools with on-campus social workers across the various school districts in Travis County. These schools are shown alongside all high school campuses and the locations of free or low-cost community mental health clinics. The map reveals that multiple high schools with embedded social workers are located in areas lacking nearby mental healthcare clinics, particularly within Del Valle, Manor, and Pflugerville school districts. In such contexts, school-based personnel may serve as a critical point of access for mental health services, mitigating geographic and transportation barriers to care. The spatial alignment of service gaps and school-based support highlights the importance of sustaining or expanding on-campus social work programs to address adolescent mental health needs where community resources are limited.

### Descriptive statistics

Table 1 presents descriptive statistics for school- and community-level variables across 24 schools. On average, 29% of schools had a social worker on site. The number of enrolled students ranged widely, from 100 to 2,899, with a mean of 1,453 (SD = 828). Substantial variation was observed in community-level sociodemographic indicators. The average proportion of residents living below the poverty line was 36% (SD = 24%), while the average unemployment rate was 51% (SD = 15%). Approximately 39% of adults lacked a high school diploma, and 23% of the population reported a disability. Single-parent households accounted for an average of 52% of all households, ranging from 18 to 95%. On average, 39% of the population was uninsured, and 60% had limited English proficiency. Additionally, 64% of residents were burdened by housing costs, and 60% identified as members of a racial or ethnic minority group. These figures reflect significant heterogeneity in both school size and community vulnerability.

**Table 1:** Descriptive statistics Travis County, Texas, for school- and community-level variables (*N* = 24 schools).

Variable	Mean	SD	Median	Min	Max	Range
Social worker on site (DV)	0.29	0.46	0.00	0.00	1.00	1.00
Number of students	1452.92	828.38	1,561	100	2,899	2,799
% below poverty line	0.36	0.24	0.29	0.08	0.73	0.65
% unemployed	0.51	0.15	0.53	0.21	0.79	0.58
% without high school diploma	0.39	0.27	0.32	0.07	0.89	0.82
% with disability	0.23	0.15	0.19	0.04	0.56	0.51
% single-parent households	0.52	0.21	0.54	0.18	0.95	0.76
% uninsured	0.39	0.25	0.38	0.06	0.86	0.80
% limited English proficiency	0.60	0.25	0.67	0.21	0.93	0.73
% housing cost burdened	0.64	0.22	0.69	0.28	0.96	0.68
% minority population	0.60	0.21	0.66	0.21	0.88	0.66

### Spatial regression results

Table 2 presents results from four spatial regression models predicting the presence of a school-based social worker. In the student-only models (Lag and Error), the number of students was not significantly associated with social worker presence. In contrast, the full demographic models identified several significant predictors. In the full Lag model, higher percentages of residents below the poverty line (*p* < 0.05), unemployed (*p* < 0.05), and with limited English proficiency (*p* < 0.05) were negatively associated with the likelihood of having a social worker, while higher rates of residents without a high school diploma (*p* < 0.05), with a disability (*p* < 0.05), and those experiencing housing cost burden (*p* < 0.05) were positively associated. The spatial error model showed a similar pattern, with significant negative associations for poverty (*p* < 0.05), unemployment (*p* < 0.05), and limited English proficiency (*p* < 0.05), and positive associations for low educational attainment (*p* < 0.05) and disability (*p* < 0.05). Marginal significance was observed for the uninsured population (*p* < 0.05) and minority composition (*p* < 0.05). These findings emphasize the spatially structured influence of community disadvantage on the distribution of school-based social work services.

**Table 2:** Spatial regression models examining demographic predictors of school-based social worker presence, Travis County, Texas (*N* = 23 schools).

Predictor	Lag model (students only)	Error model (students only)	Lag model (full)	Error model (full)
Intercept	0.130 (0.139)	0.668 (0.377)^†^	−0.124 (0.279)	0.211 (0.513)
Number of students	−0.0000016 (0.000074)	0.000042 (0.000075)	0.000057 (0.000055)	0.000032 (0.000060)
% below poverty			−2.453 (0.511)***	−2.229 (0.582)***
% unemployed			−0.615 (0.257)*	−0.731 (0.339)*
% no HS diploma			2.555 (0.782)**	2.135 (0.914)*
% with disability			1.617 (0.382)***	1.310 (0.430)**
% single-parent HH			0.370 (0.566)	0.093 (0.618)
% uninsured			0.457 (0.504)	0.941 (0.639)^†^
% limited English			−1.838 (0.472)***	−2.274 (0.606)***
% housing burdened			1.014 (0.336)**	0.492 (0.390)
% minority pop.			0.116 (0.465)	1.117 (0.607)^†^

Dependent variable: on-site social worker present (1 = Yes, 0 = No).

*p < 0.05, **p < 0.01, **p < 0.001, ^†^*p* < 0.10. All models use spatial weights based on 4-nearest neighbors from geographic coordinates.

### Model fit and spatial dependence

Table 3 reports model diagnostics and spatial fit statistics across the four regression models. Both student-only and full models exhibited strong spatial dependence, as indicated by statistically significant spatial parameters (Lag: *ρ* = 0.818 and ρ = 0.906; Error: *λ* = 0.831 and λ = 0.918; all *p* < 0.001). The full models demonstrated substantially better fit than did student size-only models, and the former produced higher log-likelihood values (Lag: 14.26; Error: 7.99) and notably lower Akaike information criterion scores (Lag: −2.51; Error: 10.00) compared to the student-only models (Akaike information criterion scores of 20.99 and 20.69, respectively). Additionally, residual variance was markedly reduced in the full models (σ^2^ = 0.0127 and 0.0214), suggesting improved explanatory power. The Lagrange Multiplier test for residual spatial autocorrelation was not significant in either model (student-only: *p* = 0.097; full: *p* = 0.344), indicating that spatial dependencies were adequately addressed by the spatial lag and error specifications. Collectively, these diagnostics support the inclusion of sociodemographic variables in improving model performance and addressing spatial structure in the data.

**Table 3:** Model fit statistics.

Statistic	Lag (students)	Error (students)	Lag (full)	Error (full)
Spatial parameter (ρ or λ)	ρ = 0.818***	λ = 0.831***	ρ = 0.906***	*λ* = 0.918***
Log likelihood	−6.50	−6.35	14.26	7.99
Akaike information criterion	20.99	20.69	−2.51	10.00
Residual variance (σ^2^)	0.0843	0.0824	0.0127	0.0214
LM test for residual spatial autocorr.	2.76 (*p* = 0.097)		0.90 (*p* = 0.344)	

## Discussion

This study provides a spatially informed assessment of adolescent mental health resources in Travis County, Texas, highlighting both geographic disparities in access to care and structural patterns in the allocation of school-based support. By integrating spatial visualization with regression modeling, the findings move beyond descriptive mapping to identify how school and neighborhood characteristics are associated with the presence of on-site social workers. By integrating school locations, student enrollment data, and proximity to low-cost or free community mental health clinics, several patterns emerge that reveal both gaps in service access and opportunities for targeted intervention. While some high schools are situated near clinics, a substantial number, particularly those in Del Valle, Manor, and Elgin school districts, are located more than two to five miles from the nearest facility. These distances may represent significant barriers for students lacking reliable transportation or experiencing socioeconomic disadvantage, creating care “deserts” (2, 7). However, access to mental health services is inherently multidimensional. While this study operationalizes access primarily as geographic proximity to clinics, other factors, such as transportation availability, service eligibility, affordability, appointment availability, and cultural or linguistic barriers, also shape whether adolescents can obtain care. As such, distance should be interpreted as one component of access rather than a comprehensive measure. Future research should incorporate these additional dimensions to better capture realized access to mental health services. Moreover, the largest high schools, enrolling over 2,000 students, are not necessarily the ones best served by proximate clinics. In some cases, the presence of on-campus social workers appears to fill critical service gaps in areas where community clinics are absent, reinforcing the need to sustain and expand school-based mental health support. This spatial mismatch between high-need areas and available services, especially in the context of recent population growth and migration shifts, highlights the importance of a coordinated, data-informed approach to adolescent mental health planning and resource allocation at the local level.

The presence of on-campus social workers emerged as a critical stopgap in areas lacking proximal mental healthcare infrastructure. However, only 29% of the 24 high schools we analyzed in Travis County had a social worker on-site, revealing a significant coverage gap. Spatial regression results showed that schools in communities with higher poverty, unemployment, and limited English proficiency were significantly less likely to have a social worker, suggesting structural and institutional barriers to resource allocation in areas of highest need (34, 35). Conversely, schools in areas with higher rates of disability, lower levels of educational attainment, and greater housing cost burdens were more likely to have on-site social workers. One possible explanation is that certain indicators of vulnerability-such as disability or educational attainment-may be more directly linked to funding formulas, service eligibility criteria, or existing program priorities. In contrast, other aspects of disadvantage, like language barriers or unemployment, might be less explicitly considered in resource allocation decisions. These findings underscore the complexity of how different forms of vulnerability are recognized and addressed within school-based mental health systems. Some of these associations may appear theoretically complex or counterintuitive. These patterns may reflect unmeasured contextual factors, differences in how vulnerability indicators are operationalized in funding or service allocation processes, or limitations related to spatial aggregation at the ZCTA level. As such, these findings should be interpreted cautiously and viewed as areas for future research rather than definitive conclusions.

These findings highlight a spatial and demographic mismatch in resource deployment that may contribute to observed patterns of health inequities among adolescents (36). These results highlight the importance of place-based and equity-focused planning in adolescent mental health service delivery. Geospatial tools can assist policymakers and school systems in pinpointing not only where services are lacking but also whether resources are properly aligned with the populations most in need. Expanding school-based mental health staffing-especially in underserved areas-along with strengthening partnerships with community providers and telehealth programs, may help reduce geographic and structural barriers to care. Incorporating spatial analysis into policy development can help ensure that limited mental health resources are allocated to where adolescent needs are the greatest (37).

Several limitations should be considered when interpreting these findings. First, given the cross-sectional and observational nature of the study, findings should be interpreted as associations rather than causal relationships. Also, the study focuses on the presence of school social workers as an indicator of school-based mental health support. While social workers play a critical role in addressing both individual and community-level factors of health, this measure does not capture other important providers, including school counselors, psychologists, or telehealth-based services. As a result, the analysis may underestimate the full scope of available mental health resources within schools. Therefore, the findings should be interpreted as reflecting one dimension of access rather than a comprehensive measure of school-based mental health service availability. In addition, the distinction between school social workers, counselors, and psychologists is not always clearly delineated in practice, particularly in resource-constrained settings where responsibilities may overlap. As behavioral health needs increase and staffing remains limited, schools may rely on multiple professional roles to address similar student needs. This functional overlap may influence how mental health services are delivered and complicates the interpretation of workforce-based measures of access. Additionally, the analysis of community mental health resources was limited to publicly available information on free or low-cost clinics and did not include private providers that may be accessible to insured populations. As a result, the study primarily reflects access to services for populations facing financial or structural barriers to care. Data on the presence of on-site social workers and clinic locations were compiled from publicly available sources and administrative information, which may be subject to reporting limitations despite efforts to verify accuracy. Furthermore, community-level characteristics were measured at the ZIP Code Tabulation Area (ZCTA) level, which may not fully capture the complexity and heterogeneity of local neighborhood conditions. ZCTAs are administrative approximations and may introduce spatial misclassification or aggregation bias, potentially affecting the precision of estimated associations. This limitation is particularly relevant when interpreting equity-related findings, as within-area variation in socioeconomic conditions may be obscured. Future research should consider using finer geographic units, such as census tracts or block groups, to more accurately capture neighborhood context. Finally, the relatively small sample size (N = 24 schools) may limit statistical power and affect the stability and precision of regression coefficient estimates. As a result, the findings should be interpreted as exploratory and hypothesis-generating rather than definitive. Small samples may increase the risk of overfitting and reduce the generalizability of results. However, the consistency in the direction and significance of key associations across spatial model specifications provides some support for the robustness of the findings. Future research using larger samples and additional geographic areas is needed to validate these patterns.

## Conclusion

These findings are consistent with an SDOH framework, in which structural disadvantage is related to both the allocation of institutional resources and the spatial accessibility of care. Adolescent mental health needs in Travis County are increasingly unmet, especially in socioeconomically vulnerable and rapidly expanding areas where access to free or low-cost clinics and on-campus social workers is limited. This study highlights significant care deserts that threaten youth well-being and academic success, with statewide telehealth services like Texas Child Health Access Through Telemedicine providing only partial support due to digital divides. To address these disparities, stakeholders should fund on-campus social workers, expand school-based health initiatives, and use spatial data to target resource deployment. Geographic mapping and spatial regression provide useful tools for identifying service gaps and guiding equitable health investments, emphasizing the need for place-based, data-driven strategies to inform efforts to improve school mental health infrastructure amidst rising adolescent mental health challenges.

## Data Availability

The original contributions presented in the study are included in the article/supplementary material, further inquiries can be directed to the corresponding author.

## References

[1] Anderson K Anderson KN Swedo EA Trinh E Ray CM Krause KH . Adverse childhood experiences during the COVID-19 pandemic and associations with poor mental health and suicidal behaviors among high school students — adolescent behaviors and experiences survey, United States, January–June 2021. Morb Mortal Wkly Rep. (2021) 71:1301–5. doi: 10.15585/mmwr.mm7141a2, 36227769 PMC9575476

[2] Golberstein E Zainullina I Sojourner A Sander MA. Effects of school-based mental health services on youth outcomes. J Hum Resour. (2024) 59:S256–81. doi: 10.3368/jhr.1222-12703r2

[3] Jones S Jones SE Ethier KA Hertz M DeGue S Le VD . Mental health, suicidality, and connectedness among high school students during the COVID-19 pandemic — adolescent behaviors and experiences survey, United States, January–June 2021. MMWR Suppl. (2021) 71:16–21. doi: 10.15585/mmwr.su7103a3, 35358165 PMC8979602

[4] Mukherjee S Gordils J. Factors associated with gun possession among high-school students in the U.S. before and during the pandemic. Psychol Rep. (2024) 128:179–97. doi: 10.1177/00332941241263750, 38913602

[5] Schlack R Peerenboom N Neuperdt L Junker S Beyer AK. The effects of mental health problems in childhood and adolescence in young adults: results of the KiGGS cohort. J Health Monitor. (2021) 6:3–19. doi: 10.25646/8863, 35146318 PMC8734087

[6] Suldo S Suldo SM Thalji-Raitano A Kiefer SM Ferron JM. Conceptualizing high school students' mental health through a dual-factor model. Sch Psychol Rev. (2016) 45:434–57. doi: 10.17105/spr45-4.434-457

[7] Merikangas KR He J Burstein M Swanson SA Avenevoli S Cui L . Lifetime prevalence of mental disorders in US adolescents: results from the National Comorbidity Survey Replication–Adolescent Supplement (NCS-A). J Am Acad Child Adolesc Psychiatry. (2010) 49:980–9. doi: 10.1016/j.jaac.2010.05.017, 20855043 PMC2946114

[8] Kessler RC Angermeyer M Anthony JC De Graaf R Demyttenaere K Gasquet I . Lifetime prevalence and age-of-onset distributions of mental disorders in the World Health Organization's world mental health survey initiative. World Psychiatry. (2007) 6:168. Available online at: https://pure.johnshopkins.edu/en/publications/lifetime-prevalence-and-age-of-onset-distributions-of-mental-diso/ (Accessed June 23, 2026). 18188442 PMC2174588

[9] Ioannou E Ravulo J Ray N. The underdeveloped role of social work in schools: a localised perspective within a Peri-urban setting. Soc Work Educ. (2023) 43:2865–79. doi: 10.1080/02615479.2023.2298331, 37339054

[10] Kanlaya D. Social worker in schools in high school students. Open J Soc Sci. (2021) 9:541–54. doi: 10.4236/jss.2021.99039

[11] Wiedebusch S. The contribution of school social work to climate change education and mental health support. Eur J Soc Work. (2024) 27:692–703. doi: 10.1080/13691457.2024.2344005

[12] Ding X Lightfoot E Berkowitz R Guz S Franklin C DiNitto DM. Characteristics and outcomes of school social work services: a scoping review of published evidence 2000–June 2022. Sch Ment Health. (2023) 15:787–811. doi: 10.1007/s12310-023-09584-z, 37359159 PMC10187493

[13] Drabenstott M Smyth RE Searle M Kirkpatrick L Labonté C. School leaders’ response to rising mental health concerns: a collaborative school-based social worker pilot. J Sch Leadersh. (2023) 33:607–32. doi: 10.1177/10526846231187569

[14] Gao D Dong Y Kong A Li X. How does perceived social support impact mental health and creative tendencies among Chinese senior high school students? Behav Sci (Basel). (2024) 14:1002. doi: 10.3390/bs14111002, 39594302 PMC11591015

[15] Dull J. A lack of workplace resources for social workers in schools. Soc Work. (2024) 69:177–84. doi: 10.1093/sw/swae008, 38390662

[16] ATD Travis County high school information (2024). Available online at: https://data.austintexas.gov/Locations-and-Maps/Travis-County-High-School-Information/b6qz-64ts/about_data (Accessed March 16, 2025)

[17] DSHS Texas health data: health profession fact sheets (2023). (Accessed March 16, 2025)

[18] NCES National Teacher and principal survey (2016). Available online at: https://nces.ed.gov/surveys/ntps/tables/ntps1516_027_s1n_04.asp (Accessed March 16, 2025)

[19] Heinrich CJ Colomer A Hieronimus M. Minding the gap: evidence, implementation and funding gaps in mental health services delivery for school-aged children. Child Youth Serv Rev. (2023) 150:107023. doi: 10.1016/j.childyouth.2023.107023, 37261333 PMC10202463

[20] Bevan SL DeWitt CC. Policy and practice innovations in school-based mental health services. Child Youth Serv Rev. (2024) 166:107970. doi: 10.1016/j.childyouth.2024.107970

[21] Gaal S Fuller M. School safety and mental health awareness: recommendations from K-12 Texas public school teachers. J Sch Health. (2024) 94:308–16. doi: 10.1111/josh.13425, 38288657

[22] Gallegos GA Schaper K Roy S Gao J. Analyzing the relationships between state school policies and absenteeism rates for 9-12th grade students in Texas-Mexico border districts. Res Educ Policy Manag. (2024) 6:255–68. doi: 10.46303/repam.2024.16

[23] Goldman B. Gracie K. Every Day Counts: Absenteeism and the Returns to Education in High-Poverty Schools, Working Paper (2024).

[24] Braveman P Egerter S Williams DR. The social determinants of health: coming of age. Annu Rev Public Health. (2011) 32:381–98. doi: 10.1146/annurev-publhealth-031210-101218, 21091195

[25] Marmot M Wilkinson R. Social Determinants of Health.Oup Oxford 2nd edn,. In: M. Marmot, R. Wilkinson, editors. Oxford, England: Oxford University Press.(2005).

[26] Braveman P Gottlieb L. The social determinants of health: it's time to consider the causes of the causes. Public Health Rep. (2014) 129:19–31. doi: 10.1177/00333549141291S206, 24385661 PMC3863696

[27] Bivand R S Wong DWS. Comparing implementations of global and local indicators of spatial association. TEST (2018) 27:716–48. doi: 10.1007/s11749-018-0599-x

[28] Bivand R. Piras G. Comparing implementations of estimation methods for spatial econometrics. J Stat Softw. (2015) 63:1–36. doi: 10.18637/jss.v063.i18

[29] Severance C. Python for Everybody: Exploring Data using python 3.Charles Severance (2016).

[30] Esri ArcGIS pro (version 3.2) [computer software] Environmental Systems Research Institute Available online at: https://www.esri.com/en-us/arcgis/products/arcgis-pro/overview (2024). (Accessed June 23, 2026).

[31] Anselin L. Spatial Regression Analysis in R: a Workbook, vol. 51. Urbana, IL: University of Illinois (2005). p. 61801.

[32] Beale CM Lennon JJ Yearsley JM Brewer MJ Elston DA. Regression analysis of spatial data. Ecol Lett. (2010) 13:246–64. doi: 10.1111/j.1461-0248.2009.01422.x, 20102373

[33] Anselin L. "Spatial regression". In: The SAGE Handbook of Spatial Analysis, vol. 1 (2009). p. 255–76.

[34] Alegría M Canino G Ríos R Vera M Calderón J Rusch D . Mental health care for Latinos: inequalities in use of specialty mental health services among Latinos, African Americans, and non-Latino whites. Psychiatr Serv. (2002) 53:1547–55. doi: 10.1176/appi.ps.53.12.1547, 12461214

[35] Flores G Fuentes-Afflick E Barbot O Carter-Pokras O Claudio L Lara M . The health of Latino children: urgent priorities, unanswered questions, and a research agenda. JAMA. (2002) 288:82–90. doi: 10.1001/jama.288.1.82, 12090866

[36] Anderson M. Perrin A. Nearly one-in-five teens can’t always finish their homework because of the digital divide. pew research center (2018) 26: 2018. Available online at: https://www.pewresearch.org/short-reads/2018/10/26/nearly-one-in-five-teens-cant-always-finish-their-homework-because-of-the-digital-divide/ (Accessed June 23, 2026).

[37] Grubesic TH Matisziw TC. On the use of ZIP codes and ZIP code tabulation areas (ZCTAs) for the spatial analysis of epidemiological data. Int J Health Geogr. (2006) 5:58. doi: 10.1186/1476-072X-5-58, 17166283 PMC1762013

[38] Substance Abuse and Mental Health Services Administration. Key Substance Use and Mental Health Indicators in the United States: Results from the 2023 National Survey on Drug Use and Health. U.S. Department of Health and Human Services (2024). Available online at: https://www.samhsa.gov/data/report/2023-nsduh-annual-national-report (Accessed June 23, 2026).

[39] U.S. Census Bureau. County Population Totals and Components of Change: 2020-2024. U.S. Department of Commerce. (2025). Available online at: https://www.census.gov/data/tables/time-series/demo/popest/2020s-counties-total.html (Accessed June 23, 2026).

[40] U.S. Department of Education, National Center for Education Statistics. The 2023-24 National Teacher and Principal Survey. National Center for Education Statistics, (2025). Available online at: https://nces.ed.gov/surveys/ntps/participants_2324.asp (Accessed June 23, 2026).

[41] Texas Education Agency. Statewide Plan for Student Mental Health: 2025–2030 Plan Refresh. Texas Education Agency (2025). Available online at: https://tea.texas.gov/families-and-students/mental-health/mental-health-and-wellness (Accessed June 23, 2026).

[42] Texas Child Mental Health Care Consortium. Texas Child Health Access Through Telemedicine (TCHATT) Program Overview and Resources. Texas Child Mental Health Care Consortium. (2025). Available online at: https://tcmhcc.utsystem.edu/tchatt/ (Accessed June 23, 2026).

